# Enhancement of In Vitro Production of Volatile Organic Compounds by Shoot Differentiation in *Artemisia spicigera*

**DOI:** 10.3390/plants10020208

**Published:** 2021-01-22

**Authors:** Saeedeh Ghorbani, Morteza Kosari-Nasab, Sepideh Mahjouri, Amir Hossein Talebpour, Ali Movafeghi, Filippo Maggi

**Affiliations:** 1 Department of Plant Sciences, Faculty of Natural Sciences, University of Tabriz, Tabriz 5166616471, Iran; s.ghorbani96@stu.gu.ac.ir (S.G.); kosarinasabm@tbzmed.ac.ir (M.K.-N.); 2 Drug Applied Research Center, Tabriz University of Medical Sciences, Tabriz 5165665811, Iran; 3 Department of Biological Sciences, Faculty of Basic Sciences, Higher Education Institute of Rab-Rashid, Tabriz 5155958316, Iran; mahjouri@raberashidi.ac.ir; 4 Research Center for Agriculture and Natural Resources, East Azerbaijan, Tabriz 5153715898, Iran; a.talebpour@areeo.ac.ir; 5 School of Pharmacy, University of Camerino, 62032 Camerino, Italy

**Keywords:** *Artemisia spicigera*, tissue culture, plantlet regeneration, volatile organic compounds

## Abstract

Callus initiation, shoot formation and plant regeneration were established for *Artemisia spicigera*, a traditional medicinal plant growing in Armenia, Middle-Anatolia and Iran, and producing valuable volatile organic compounds (VOCs) that are mostly represented by monoterpenoids. Optimal callus initiation and shoot production were obtained by culture of hypocotyl and cotyledon explants on MS medium comprising 0.5 mg L^−1^ naphthalene acetic acid (NAA) and 0.5 mg L^−1^ 6-benzyladenine (BA). Consequently, the shoots were transferred onto the MS media supplemented with 1 mg L^−1^ of indole-3-butyric acid (IBA) or 1 mg L^−1^ of NAA. Both types of auxin induced root formation on the shoots and the resulting plantlets were successfully grown in pots. The production of VOCs in callus tissues and regenerated plantlets was studied by gas chromatography–mass spectrometry (GC-MS) analysis. Although the potential of undifferentiated callus to produce VOCs was very low, an increased content of bioactive volatile components was observed at the beginning of shoot primordia differentiation. Intriguingly, the volatiles obtained from in vitro plantlets showed quantitative and qualitative variation depending on the type of auxins used for the rooting process. The acquired quantities based on total ion current (TIC) showed that the regenerated plantlets using 1 mg L^−1^ NAA produced higher amounts of oxygenated monoterpenes such as camphor (30.29%), *cis*-thujone (7.07%), and 1,8-cineole (6.71%) and sesquiterpene derivatives, namely germacrene D (8.75%), bicyclogermacrene (4.0%) and spathulenol (1.49%) compared with the intact plant. According to these findings, in vitro generation of volatile organic compounds in *A. spicigera* depends on the developmental stages of tissues and may enhance with the formation of shoot primordia and regeneration of plantlets.

## 1. Introduction

Plant tissue culture is an important technique applied in different areas of research for micropropogation, secondary metabolites production, and toxicological studies [1,2,3]. In vitro regeneration can save endangered and superior genotypes and also improves the chemical profiles of medicinal plants [4]. Therefore, the application of tissue culture for studying the regeneration potential and changes in the chemical composition is very important to improve the production of natural products obtainable from valuable medicinal and aromatic plants.

*Artemisia* L. (Asteraceae) is a large, diverse, and widely distributed genus with more than 500 species. This genus comprises of bitter, aromatic herbs or shrubs, which are famed for the production of volatile organic compounds (VOCs) of medicinal value [5,6]. *Artemisia* species have been reported to be useful in the therapy of a number of ailments including malaria, cancer, hepatitis, and also for different diseases caused by infectious microorganisms [7]. Hence, an extensive attention in biotechnological researches of the genus has been emerged over the past decades [8,9]. Different approaches have been improved to enhance pharmaceutically important metabolites through in vitro cultures of *Artemisia* [10].

*Artemisia spicigera* C. Koch is an aromatic perennial herb growing in Armenia, Middle-Anatolia and Iran [11]. This species has antiseptic and stomachic properties and is traditionally advised for the treatment of skin infectious complaints in Turkey [7]. The volatile oil of *A. spicigera* contains mainly monoterpenoids, with 1.8-cineole (56.8%), camphor (20.2%), and camphene (4.9%) as the major constituents [11]. The main aim of the current study was to develop an easy procedure for tissue culture and plant regeneration of *A. spicigera* using hypocotyl and cotyledon explants. Afterwards, the potential of callus tissues and regenerated plantlets for the production of VOCs has been assessed using GC-MS analysis.

## 2. Materials and Methods

### 2.1. In Vitro Culture Conditions

The seeds of *A. spicigera* were obtained from the Central Botanical Garden of East Azerbaijan of Iran. Seeds were immersed in a 5% (*v*/*v*) sodium hypochlorite solution for 15 min and rinsed several times prior to be germinated onto the growth-regulator-free solidified Murashige and Skoog (MS) medium. Cotyledon and hypocotyl explants of 7-day-old seedlings were employed for callus induction experiments.

The explants with a 3–5 mm length were placed on MS medium supplemented with the combinations of naphthalene acetic acid (NAA) (0.5 and 1 mg L^−1^) and 6-benzyl adenine (BA) (0.5 and 1 mg L^−1^) at 25 ± 2 °C under 16/8 light/dark period. Subcultures were performed at intervals of four weeks using the same media. For evaluation of growth parameters, 2-month-old callus tissues were separated from the medium and fresh weights (FW) were evaluated. The calli were then dried at 50 °C for 1 day, afterwards the callus dry weights (DW) were recorded. For root formation, shoots regenerated in the medium containing 0.5 + 0.5 mg L^−1^ of NAA + BA that showed proper growth characteristics were selected. Individual shoots were cut from the 2-month-old calli and transferred onto MS media containing 1 mg L^−1^ of NAA or 3-indolebutyric acid (IBA). One group of shoots was incubated on basal MS media without growth regulators as control set. After two weeks of culture, the morphologically uniform plantlets with established roots were planted in plastic cups containing autoclaved perlit:sand (1:1) for acclimatization.

### 2.2. Preparation of the Extracts

Two-month-old undifferentiated calli, two-month-old calli with shoot primordia and shoots of micropropagated plantlets from cotyledon as well as aerial parts of the collected plants (from the Central Botanical Garden of East Azerbijan, Iran) were submitted to the solvent extraction of volatile products. An amount of 3 g of fresh samples was crushed in 20 mL of *n*-hexane for 2.5 h [12]. The extracts were dehydrated over anhydrous sodium sulfate (concentrated to 1 mL), and kept in sealed vials at 4 °C until GC-MS analysis.

### 2.3. Analysis of VOCs

Analysis of VOCs was achieved by means of a Shimadzu GC-MS-QP 5050A (Shimadzu Corporation, Kyoto, Japan). Separation of analytes was succeeded on a 60 m × 0.25 mm i.d. DB-1 capillary column coated with a film (0.25 μm f.t.,) of dimethylpolysiloxane (J&W Scientific, Folsom, CA, USA) using helium (99.99%) as carrier gas at a flow rate of 1 mL min^−1^. The injection of 0.2 µL of the *n*-hexane extract was carried out in the split mode with a split ratio of 1:5. The rising column temperature was programmed from 50 °C to 300 °C at a ramp rate of 3 °C min^−1^. The temperatures of injector and transfer line detector were optimized at 280 and 310 °C, respectively. Mass spectra were attained at ionization energy of 70 eV and a mass/charge scan range of 30–600 *m/z* at a sampling rate of 2 scans s^−1^. Constituents were recognized by comparison of their retention indices, experimentally determined using a mixture of linear C_7_–C_30_ alkanes (Supelco, Bellefonte, PA, USA), and mass spectra with those stored in Adams and NIST 20 and WILEY 12 databases. The percentages of analytes were taken from the GC-MS chromatograms without using response factors since the most abundant components belong to the same group (monoterpenoids). Based on literature papers [13], the MS response to these compounds is not far from that of flame-ionization detection (FID).

### 2.4. Data Analysis

The data were collected after 8 weeks for shoot regeneration and 2 weeks for rooting experiments. The experiments were conducted with four replicates and seven explants in each replicate using a completely randomized design. Analysis of variance (ANOVA) was applied to examine the data sets and comparing the treatment means were reported based on the Duncan’s multiple range test (*p* ≤ 0.05). The standing data for all assessments were average values from the four separate experiments and were equated using standard error of the means (SE).

## 3. Results and Discussion

### 3.1. Callus Induction and In Vitro Regeneration

Callus initiation was induced by the applied concentrations of plant growth regulators (0.5 + 0.5 and 1 + 1 mg L^−1^ of NAA + BA) in both hypocotyl and cotyledon explants. No callus formation occurred on the basal hormone-free medium. There was no significant difference in fresh weights of the calli formed on media with altered combinations of NAA and BA compared to the control sample. However, the dry weight of calli originated from both hypocotyl and cotyledon explants in the concentration of 0.5 + 0.5 mg L^−1^ NAA + BA was significantly different when compared with the control medium. These results indicated that the medium containing 0.5 mg L^−1^ of NAA + 0.5 mg L^−1^ of BA is more suitable for the establishment and growth of callus tissues of *A. spicigera* in comparison with other treatments (Table 1). Without further treatments, shoot initiation occurred on the calli within 4 weeks following culture.

**Figure 1:**
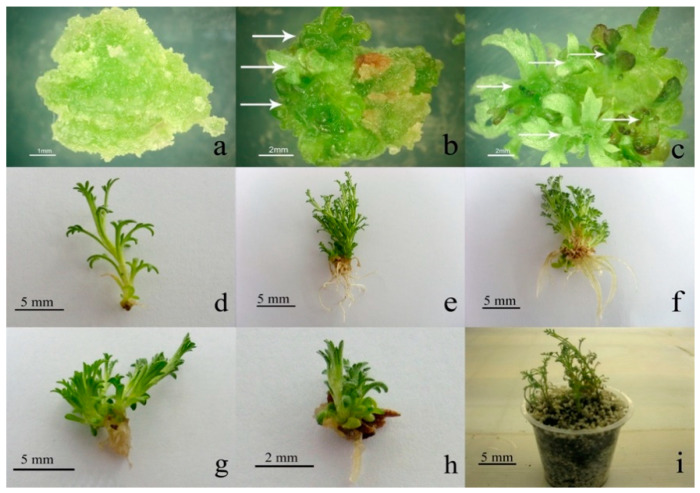
Different stages of callus formation and plantlet regeneration in *Artemisia spicigera*. Callus induction from cotyledon explants on MS medium containing 1 mg L^−1^ of NAA + BA (**a**). Shoot bud formation (arrows) on the callus tissues originated from cotyledon explants after 30 days of culture on MS medium with 1 (**b**) and 0.5 mg L^−1^ of NAA + BA (**c**). Rooting of in vitro regenerated shoots originated from the hypocotyl explants on control MS medium without growth regulators (**d**), containing 1 mg L^−1^ of NAA (**e**) and 1 mg L^−1^ of IBA (**f**). Root formation of in vitro regenerated shoots originated from cotyledon explants by using 1 mg L^−1^ of NAA (**g**) and 1 mg L^−1^ of IBA (**h**). Acclimatization of the regenerated plantlets with developed roots in perlit:sand (1:1) (**i**).

Shoot proliferation increased after the first subculturing of calli on the same media. Shoot production on the hypocotyl and cotyledon explants was not influenced by different levels of the growth regulators (Table 1; Figure 1). De novo shoot organogenesis in tissue culture is an essential step in most plant micropropagation systems. In the present work, we report a medium appropriate for rapid *A. spicigera* shoot induction following callus formation. BA, as a synthetic cytokinin, in combination with fitting auxins has been frequently used for callus culture and shoot proliferation of various plant species [14,15]. As an effective plant growth regulator, it modulates several biotechnological processes and influences several stages of plant growth and development. Our results suggest that BA might be an effective cytokinin in shoot proliferation of *A. spicigera*. This finding is consistent with the outcomes of preceding reports on the shoot proliferation of other *Artemisia* species such as *A. alba* [16], *A. absinthium* [17], and *A. annua* [18].

Regenerated shoots were excised and shifted to the rooting medium comprising NAA or IBA (1 mg L^−1^) and medium without growth regulators as a control. After approximately 7 days, roots became visible at the base of the shoots. In the case of hypocotyl explants, the use of both NAA and IBA significantly improved the root number/length compared to the control medium (Table 2, Figure 1d–f), but none of the growth regulators could significantly increase the root number/length at the shoots originated from cotyledon explants in comparison to the control (Table 2; Figure 1g,h). IBA treatment could increase the shoot number on hypocotyl originated plantlets in comparison to the control (Table 2).

Accordingly, hypocotyl can be a more suitable explant for in vitro regeneration of *A. spicigera* in the medium supplemented with NAA and IBA. The rooted plantlets were moved to pots holding sterile perlit:sand (1:1) for hardening and located in culture room conditions (25 ± 2 °C). All of the micropropagated plants grew well and did not show any apparent morphological abnormality during the observation period (Figure 1i).

So far, some regeneration procedures have been undertaken for in vitro propagation of *Artemisia* species for different aims [17,19,20]. Actually, effects of explant origin and growth regulators were examined during organogenesis steps. However, the optimum micropropagation conditions varied depending on the species. Thus, setting up a competent system for micropropagation of *A. spicigera* as a valuable medicinal plant seemed to be essential. In the present study, a simple, reliable, and fast regeneration protocol was successfully developed for *A. spicigera*, which provides a basis for further investigations on other members of the genus.

### 3.2. Identification of VOC in Different Tissue Extracts

The identified VOCs by GC-MS analysis are presented in Table 3. Based on the data, the whole plant produced considerable amounts of VOCs. In total, 27 VOCs were identified in the volatile profile of the whole plant, among which oxygenated monoterpenes including camphor, *cis*-thujone, and 1,8-cineole constituted the main chemical class. This outcome is in line with the former reports regarding the concept that the volatile oil constitutes in *A. spicigera* may possibly change in relation to the geographical location, climatic condition, soil type and regional cultivar [11,21]. In addition, secondary metabolites content of plants may vary noticeably at different physiological/developmental stages [7].

By contrast, the potential of callus tissues in the production of VOCs was very low. In undifferentiated callus tissues only α-pinene, *cis*-thujone, and camphor were identified in low quantities as bioactive compounds. Intriguingly, with the emergence of morphological signs of the shoot initiation on the calli, some VOCs were detected by GC-MS in the callus tissues. This outcome reveals the association between in vitro shoot induction and volatile compounds’ production. Alternatively stated, in vitro metabolic pathways of volatiles in *A. spicigera* are developmentally organized. These results are also in fair agreement with previous reports in other plant species [22].

The volatiles acquired from in vitro plantlets displayed qualitative variation depending on the type of auxin used in the culture media. The shoots of the plants regenerated on the media supplemented with 1 mg L^−1^ of IBA produced simply two VOCs including camphor and *cis*-thujone. In comparison, the regenerated shoots of the plantlets in presence of NAA produced large amounts of oxygenated monoterpenes such as camphor, *cis*-thujone, and 1,8-cineole as well as sesquiterpene derivatives, namely, germacrene D, bicyclogermacrene, and spathulenol (Table 4). As a matter of fact, the auxins applied in the adventitious root formation media, not only governed the in vitro differentiation events but also regulated the biosynthesis of VOCs. The impact of the plant growth regulators on the volatile composition of in vitro developed shoots of various plants was previously studied. Taken together, although the VOC composition of in vitro developed shoots was not qualitatively influenced by cytokinin composition of culture media [23,24], it differed markedly depending on the type of auxin in the medium [22]. Up to now, the mechanism of effects of auxins on the biosynthetic pathways of VOCs has not been well documented. Thus, additional investigations are needed to find out any relationship between the types and quantities of auxins and those of VOCs.

## 4. Conclusions

Our work conclusively showed that *A. spicigera* has notable potential for in vitro production of bioactive VOCs. In fact, the in vitro production of VOCs revealed to be correlated with the tissues developmental stages. The content of VOCs detected by GC-MS in callus tissues enhanced with the formation of shoot primordia and regeneration of plantlets. Accordingly, micropropagated plantlets of *A. spicigera* could produce higher amounts of VOCs compared to callus of this species. The auxins added to the rooting media, not only regulated the in vitro root initiation processes, but also influenced the quality of VOCs.

## Figures and Tables

**Table 1:** The effect of different concentrations of plant growth regulators on growth and development parameters of callus tissues in *Artemisia spicigera*.

Treatment	Shoot Number		Fresh Weight (mg)		Dry Weight (mg)	
	Hypocotyl	Cotyledon	Hypocotyl	Cotyledon	Hypocotyl	Cotyledon
Control	0 ^b^	0 ^b^	0 ^a^	0 ^a^	0 ^b^	0 ^b^
NAA + BA (0.5 mg L^−1^)	5.75 ± 2.70 ^a^	8.50 ± 4.06 ^a^	857 ± 611 ^a^	650 ± 272 ^a^	106 ± 9 ^a^	103 ± 75 ^a^
NAA + BA (1 mg L^−1^)	4.75 ± 2.80 ^a^	2.87 ± 1.68 ^a^	363 ± 140 ^a^	613 ± 322 ^a^	23 ± 4 ^ab^	32 ± 16 ^ab^

Means ± SE followed by same letters are not significantly different (*p* < 0.05). In control media, the explants were degenerated and died; therefore, their weights were reported as 0.

**Table 2:** Comparison of the effects of NAA and IBA on rooting parameters and shoot traits of *Artemisia spicigera*.

Treatment	Root Number		Root Length (mm)		Shoot Number		Shoot Length (mm)	
	Hypocotyl	Cotyledon	Hypocotyl	Cotyledon	Hypocotyl	Cotyledon	Hypocotyl	Cotyledon
Control	0.22 ± 0.04 ^b^	0.075 ± 0.15 ^b^	0.48 ± 0.05 ^c^	0.25 ± 0.05 ^c^	0.88 ± 0.05 ^b^	1.40 ± 0.65 ^a^	5.05 ± 0.20 ^a^	6.22 ± 1.43 ^a^
NAA (1 mg L^−1^)	5.50 ± 2.38 ^a^	2.24 ± 0.27 ^ab^	10.04 ± 5.17 ^ab^	3.15 ± 0.90 ^abc^	1.01 ± 0.28 ^b^	1.62 ± 0.77 ^a^	17.35 ± 10.67 ^a^	7.80 ± 2.55 ^a^
IBA (1 mg L^−1^)	5.30 ± 1.02 ^a^	0.37 ± 0.04 ^b^	11.70 ± 4.50 ^a^	1.60 ± 0.20 ^bc^	4.80 ± 2.28 ^a^	1.46 ± 0.07 ^a^	14.97 ± 6.21 ^a^	3.95 ± 0.23 ^a^

Means ± SE followed by same letters are not significantly different (*p* < 0.05).

**Table 3:** Volatile organic compounds (VOCs) identified in different extract samples of *Artemisia spicigera*.

Compound	Classification	RI ^1^	Lit. RI ^2^	Similarity (%) ^3^	Whole Plant (%) ^4^	Undifferentiated Calli (%)	Calli with Shoot Primordia (%)	Micropropagated Plantlet Using IBA (%)	Micropropagated Plantlet Using NAA (%)
Hexanal	Carbonyl compounds	794	801	97	0.13	-	-	-	-
α-Pinene	Monoterpene hydrocarbons	933	939	93	-	1.65	-	-	-
Camphene	Monoterpene hydrocarbons	952	954	97	0.40	-	-	-	-
Sabinene	Monoterpene hydrocarbons	975	969	93	0.12	-	-	-	-
α-Terpinene	Monoterpene hydrocarbons	1018	1017	95	0.21	-	-	-	-
*p*-Cymene	Monoterpene hydrocarbons	1027	1024	94	0.15	-	-	-	-
1,8-Cineole	Oxygenated monoterpenes	1033	1031	97	17.85	-	7.14	-	6.71
γ-Terpinene	Monoterpene hydrocarbons	1059	1059	95	0.37	-	-	-	-
*cis*-Sabinene hydrate	Oxygenated monoterpenes	1075	1070	90	0.47	-	-	-	0.56
*cis*-Thujone	Oxygenated monoterpenes	1103	1102	94	18.59	1.37	9.55	9.07	7.07
*trans*-Thujone	Oxygenated monoterpenes	1114	1112	95	4.84	-	5.08	-	5.23
Isothujol	Oxygenated monoterpenes	1133	1138	86	0.63	-	-	-	-
Camphor	Oxygenated monoterpenes	1144	1146	97	29.59	2.28	18.79	58.79	30.29
*trans*-Verbenol	Oxygenated monoterpenes	1150	1144	88	1.25	-	-	-	-
Pinocarvone	Oxygenated monoterpenes	1162	1164	90	-	-	-	-	1.55
Borneol	Oxygenated monoterpenes	1165	1169	97	4.03	-	-	-	-
Terpinen-4-ol	Oxygenated monoterpenes	1189	1177	95	1.39	-	-	-	-
Myrtenal	Oxygenated monoterpenes	1193	1195	91	0.75	-	-	-	0.82
Myrtenol	Oxygenated monoterpenes	1194	1194	86	0.15	-	-	-	0.95
*trans*-Piperitol	Oxygenated monoterpenes	1208	1208	90	1.04	-	-	-	-
Carvone	Oxygenated monoterpenes	1243	1243	96	0.36	-	-	-	-
Piperitone	Oxygenated monoterpenes	1253	1252	86	1.10	-	-	-	-
Chrysanthenyl acetate	Oxygenated monoterpenes	1265	1265	91	4.87	-	-	-	-
*p*-Cymen-7-ol	Oxygenated monoterpenes	1287	1290	93	0.21	-	-	-	-
Bornyl acetate	Oxygenated monoterpenes	1289	1288	96	2.20	-	-	-	-
Carvacrol	Oxygenated monoterpenes	1298	1299	88	0.22	-	-	-	-
β-Elemene	Sesquiterpene hydrocarbons	1391	1390	90	-	-	-	-	0.77
Germacrene D	Sesquiterpene hydrocarbons	1480	1485	95	1.29	-	5.24	-	8.75
Bicyclogermacrene	Sesquiterpene hydrocarbons	1494	1500	91	0.15	-	-	-	4.00
Spathulenol	Oxygenated sesquiterpenes	1575	1578	95	0.56	-	8.63	-	1.49

^1^ Linear retention index on the DB-1 capillary column, experimentally determined using a mixture of n-alkanes. ^2^ Retention index value taken from Adams library. ^3^ Match quality percentage of mass spectrum fragmentation respect to those stored in commercial libraries. ^4^ Percentage values were obtained from peak areas in the GC-MS chromatograms without using correction factors.

**Table 4:** The content of different classes of identified volatile organic compounds in whole plant and micropropagated plantlet using NAA.

Class of Compounds	Whole Plant (%) ^1^	Micropropagated Plantlet Using NAA (%) ^1^
Carbonyl compounds	0.13	-
Monoterpene hydrocarbons	1.25	-
Oxygenated monoterpenes	89.54	53.18
Sesquiterpene hydrocarbons	1.44	13.52
Oxygenated sesquiterpenes	0.56	1.49

^1^ Percentage values were obtained from peak areas in the GC-MS chromatograms without using correction factors.

## Data Availability

Data available on request.
